# New method for retrospective study of hemodynamic changes before and after aneurysm formation in patients with ruptured or unruptured aneurysms

**DOI:** 10.1186/1471-2377-13-166

**Published:** 2013-11-06

**Authors:** Wei-Jie Le, Yue-Qi Zhu, Ming-Hua Li, Lei Yan, Hua-Qiao Tan, Shi-Min Xiao, Ying-Sheng Cheng

**Affiliations:** 1 Department of Radiology, The Sixth Affiliated People’s Hospital, Medical School of Shanghai Jiao Tong University, No. 600, Yi Shan Road, Shanghai 200233, China

**Keywords:** Cerebral aneurysm, Hemodynamics, Wall shear stress

## Abstract

**Background:**

Prospective observation of hemodynamic changes before and after formation of brain aneurysms is often difficult. We used a vessel surface repair method to carry out a retrospective hemodynamic study before and after aneurysm formation in a ruptured aneurysm of the posterior communicating artery (RPcomAA) and an unruptured aneurysm of the posterior communicating artery (URPcomAA).

**Methods:**

Arterial geometries obtained from three-dimensional digital subtraction angiography of cerebral angiograms were used for flow simulation by employing finite-volume modeling. Hemodynamic parameters such as wall shear stress (WSS), blood-flow velocity, streamlines, pressure, and wall shear stress gradient (WSSG) in the aneurysm sac and at the site of aneurysm formation were analyzed in each model.

**Results:**

At “aneurysm” status, hemodynamic analyses at the neck, body, and dome of the aneurysm revealed the distal aneurysm neck to be subjected to the highest WSS and blood-flow velocity, whereas the aneurysm dome presented the lowest WSS and blood-flow velocity in both model types. More apparent changes in WSSG at the aneurysm dome with an inflow jet and narrowed impaction zone were revealed only in the RPcomAA. At “pre-aneurysm” status, hemodynamic analyses in both models showed that the region of aneurysm formation was subjected to extremely elevated WSS, WSSG, and blood-flow velocity.

**Conclusions:**

These data suggest that hemodynamic analyses in patients with ruptured or unruptured aneurysms using the vessel surface repair method are feasible, economical, and simple. Our preliminary results indicated that the arterial wall was subjected to elevated WSS, WSSG and blood-flow velocity before aneurysm generation. However, more complicated flow patterns (often with an inflow jet or narrowed impaction zone) were more likely to be observed in ruptured aneurysm.

## Background

Cerebral aneurysms are known to cause lethal subarachnoid hemorrhage. Cerebral aneurysms occur more frequently in the posterior communicating segment of the internal carotid artery than elsewhere, accounting for nearly 30% of the incidence [1,2].

Hemodynamic status is recognized as one of the most important factors responsible for the growth, development, and rupture of aneurysms [3]. Hemodynamic variables such as flow pattern, wall shear stress (WSS) and wall shear stress gradient (WSSG) have been hypothesized to be the causes of aneurysms [4].

A brain aneurysm is a type of cerebral vascular disease associated with deficiencies in the arterial wall. The formation or growth of an aneurysm is caused by remodeling of the arterial wall under long-term, complicated hemodynamic actions [5,6]. Consequently, prospective observation of hemodynamic changes during the development of brain aneurysms is extremely difficult and often restricted because it is time-consuming, expensive, and ethical issues are involved [7]. Often, there are no data before aneurysm growth for the many patients with ruptured or unruptured aneurysms to enable a prospective comparative study to be carried out. Therefore, development of a more economic and efficient method to study hemodynamic changes before and after aneurysm growth, in ruptured or unruptured aneurysms, is crucial.

We used a vessel surface repair method to carry out a retrospective hemodynamic study before and after aneurysm formation in a ruptured aneurysm of the posterior communicating artery (RPcomAA) and an unruptured aneurysm of the posterior communicating artery (URPcomAA). We then investigated the feasibility and efficacy of this method.

## Methods

### Ethical approval of the study protocol

The study protocol was approved by the Ethics Committee of the Sixth Affiliated People’s Hospital of Shanghai Jiao Tong University (No. 600, Yi Shan Road, Shanghai, China). Both study subjects provided written informed consent to participate in the study.

### Patient data

#### RPcomAA

A 62-year-old male presented to our department with a sudden headache of 8-h duration accompanied by nausea and vomiting (Hunt–Hess grade II). CT of the brain demonstrated subarachnoid hemorrhage (Fisher grade III). Cerebral magnetic resonance angiography (MRA) showed a left posterior communicating aneurysm. Angiography confirmed the diagnosis, and the aneurysm received subsequent coil embolism treatment.

#### URPcomAA

A 48-year-old male presented to our department with left facial numbness of 1-week duration. He had a history of hypertension. CT of the brain showed a lacunar infarct of the basal ganglia. Magnetic resonance imaging (MRI) of the brain showed infarction of the brainstem. Cerebral MRA revealed a left posterior communicating aneurysm. This patient underwent angiography and subsequent coil embolism for this unruptured aneurysm.

### Angiographic data

Endovascular angiography was conducted under local anesthesia using a right femoral approach. Selective catheterization of the affected side of the internal carotid artery was carried out using a 5-F vertebral catheter (Terumo, Tokyo, Japan). Both patients underwent two-dimensional (2D) cerebral angiography and rotational angiography with 3D image reconstruction using a biplanar angiographic unit (Artis zee biplane system; Siemens, Munich, Germany).

### Geometric analyses

Mimics software (Mimics 11; Materialise, Leuven, Belgium) was used to reconstruct 3D geometric models of the aneurysm and local vasculature from sections of serial image data. Geometric measurements were obtained from the 3D reconstructed models using Mimics, Geomagic studio (Geomagic, Research Triangle Park, NC, USA) and SolidWorks (Dassault Systèmes SolidWorks Corporation, Concord, MA, USA).

Briefly, the central lines of the parent vessel and aneurysm were determined. The cross-sections of the neck and widest region perpendicular to the centerline were obtained. Several 3D parameters were obtained (maximum aneurysm diameter (D); aneurysm height (H); diameter of the aneurysm neck (N)) and three non-dimensional geometric parameters (height-to-neck (H/N); diameter-to-height (D/H); diameter-to-neck (D/N)) measured [8].

### Computational fluid dynamics (CFD) analyses

CFD analyses were carried out using data collected form three-dimensional digital subtraction angiography (3D-DSA). Blood flow in the reconstructed models was simulated on the basis of the unsteady 3D Navier–Stokes equations using the finite-element method with ANSYS software (Adina R&D; Watertown, MA, USA).

For analyses of hemodynamics at the “pre-aneurysm” status, we used the variance and mean curvature calculation method to recover the parent artery back to before the aneurysm was formed. During the repair processes, point cloud processing in reverse engineering software (Geomagic) was used to calculate the filling points in the affected area based on the complex of the boundary contour of the data model. This software was then used to construct the surface of the affected area and, lastly, to measure the hemodynamic parameters of points at the constructed surface. CFD analyses were conducted for each model for one cardiac cycle with 100 time steps per cycle.

After setting pre-processing parameters (Additional file 1), CFX 11.0 was used to analyze the streamlines, velocity field, arterial wall surface pressure field, WSS, and WSSG in the aneurysm sac at the aneurysm status and in the region of the parent artery before aneurysm formation at the pre-aneurysm status. All hemodynamic parameters were measured at the peak of the systolic phase. For quantitative analyses of the aneurysm, we selected sites at the cross-sections (the dome, body, and neck) of the aneurysm to calculate the values of pressure, blood flow velocity, and WSS. For further quantitative analyses of hemodynamics at the pre-aneurysm status, we initially chose a section perpendicular to the parent artery at the region of aneurysm formation. We then measured pressure, blood flow velocity, and WSS at four points and compared them with other points at the adjacent normal artery.

Patterns of blood flow in the aneurysm sac were also investigated. They were divided into four flow types as reported by Cebralet et al. That is: type I (no change in direction of the inflow jet with a single associated vortex; type II (no change in direction of the inflow jet with multiple associated vortices, and no change in the number of vortices during the cardiac cycle); type III (change in direction of the inflow jet with creation of a single vortex); and type IV (change in direction of the inflow jet with creation or destruction of multiple vortices) [9]. The width of the inflow jet and impaction zone during the cardiac cycle was also determined. An narrowed inflow jet or impaction zone was defined as <50% of the transverse diameter or contour of the maximum cross-section parallel to the streamlines in the aneurysm sac.

## Results

### Geometric analyses

**Figure 1:**
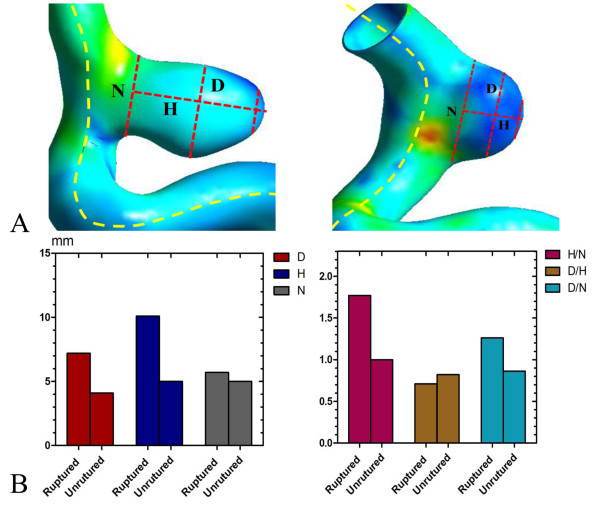
**Geometric parameters measurement and results in the RPcomAA and URPcomAA. A**, How to measure dimensional geometric parameters such as height (H), diameter (D), and neck (N) (schematic). **B**, Results of measurements of dimensional parameters and non-dimensional parameters.

The mean values of D, H, and N in the RPcomAA were (in mm) 7.20, 10.1, and 5.70 compared with 4.10, 5.0, and 5.0 in the URPcomAA, respectively. With respect to non-dimensional parameters, H/N, D/H, and D/N were 1.77, 0.71, and 1.26 in the RPcomAA, and 1.00, 0.82 and 0.86 in the URPcomAA, respectively (Figure 1).

### Hemodynamic analyses at the aneurysm status

Based on Doppler ultrasound measurements, the blood flow velocity of internal carotid artery in the RPcomAA and URPcomAA patients was 67.6 cm/s and 58.3 cm/s at the systolic phase, and 26.2 cm/s and 20.2 cm/s at the diastolic phase, with a mean heart rate of 79 bpm and 75 bpm, respectively.

**Figure 2:**
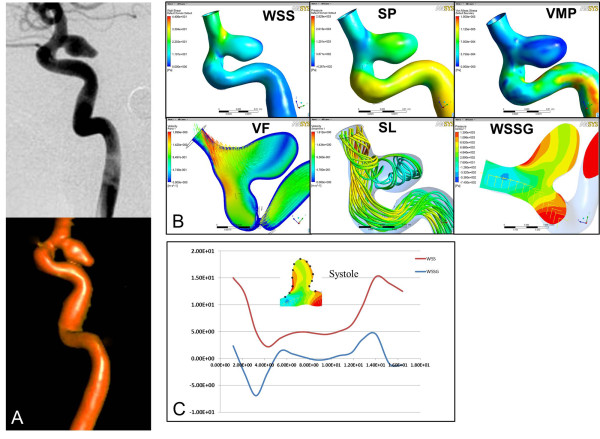
**Hemodynamic analyses of the RPcomAA at the aneurysm status. A**, Two-dimensional-DSA and 3D-DSA reconstruction images of the RPcomAA. **B**, Hemodynamic analyses in terms of WSS, pressure, velocity field, streamlines, and WSSG show elevated WSS and low pressure at the distal aneurysm neck, and decreased WSS and elevated pressure at the aneurysm dome. A complex blood flow pattern is observed in the aneurysm sac with a narrowed inflow jet and impaction zone. **C**, Quantification along the longitudinal section of the aneurysm wall shows significant changes in the distribution of WSS and WSSG at the peak of the systolic period. WSS, wall shear stress; P, pressure VMP, von Mises pressure; VF, velocity field; SL, streamlines; WSSG, wall shear stress gradient.

**Figure 3:**
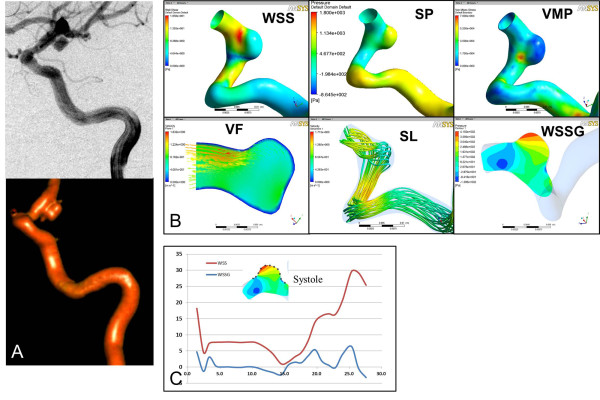
**Hemodynamic analyses of the URPcomAA at the aneurysm status. A**, Two-dimensional-DSA and 3D-DSA reconstruction images of the URPcomAA. **B**, Hemodynamic analyses in terms of WSS, pressure, velocity field, streamlines, and WSSG shows high WSS and low pressure at the distal aneurysm neck, and decreased WSS and elevated pressure at the aneurysm dome. **C**, Quantification along the longitudinal section of the aneurysm wall shows changes in the distribution of WSS and WSSG at the peak of the systolic period. WSS, wall shear stress; P, pressure VMP, von Mises pressure; VF, velocity field; SL, streamlines; WSSG, wall shear stress gradient.

**Figure 4:**
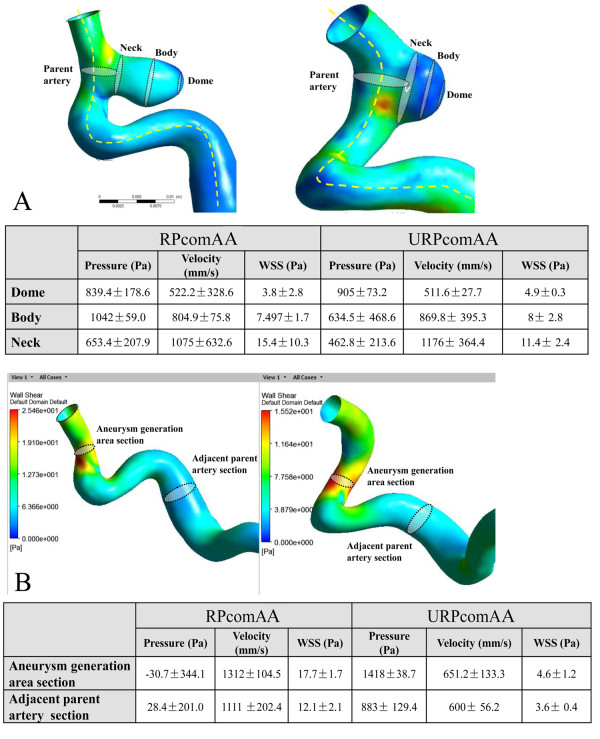
**WSS and blood flow velocity measurement in the RPcomAA and URPcomAA at the aneurysm and pre-aneurysm status. A**, How to choose sections of the neck, body, and dome of an aneurysm (schematic). Hemodynamic data analyses show that the aneurysm neck is subject to elevated WSS and blood-flow velocity, whereas the dome is subjected to the lowest WSS and blood-flow velocity. **B**, How to choose sections at the area of aneurysm formation and adjacent parent artery (schematic). Hemodynamic data analyses show that the area of aneurysm formation is subject to extremely high WSS and blood-flow velocity compared with the adjacent parent artery.

In both patients, a similar decrease of WSS and increase in pressure were observed from the aneurysm neck to the dome in the RPcomAA (WSS, 15.40 ± 10.31 Pa at the neck *vs*. 3.847 ± 2.843 Pa at the dome; pressure, 653.4 ± 207.9 Pa at the neck *vs*. 839.4 ± 178.6 Pa at the dome) and in the UPcomAA (WSS. 11.38 ± 2.417 Pa at the neck *vs*. 4.85 ± 0.2772 Pa at the dome; pressure: 462.8 ± 213.6 Pa at the neck *vs*. 905 ± 73.18 Pa at the dome). The velocity field and streamlines indicated complex turbulent blood flow in the RPcomAA compared with the normal ordered vortex flow observed in the URPcomAA. High velocity of blood flow often appeared at the distal end of the neck and decreased from the neck to the dome in the RPcomAA (1075 ± 632.6 *vs*. 522.2 ± 328.6 m/s) and URPcomAA (1176 ± 364.4 *vs*. 511.6 ± 27.7 m/s). A more significant change in WSSG was observed in the RPcomAA than in the URPcomAA (Figures 2, 3 and 4A).

We observed an narrowed inflow jet acting directly on the distal aneurysm wall with a narrowed impaction zone, where blood flow velocity was decreased significantly in the RPcomAA. However, no narrowed inflow jet had formed and a wide impaction zone was observed in the URPcomAA (Figure 2B).

### Hemodynamic analyses at the pre-aneurysm status

**Figure 5:**
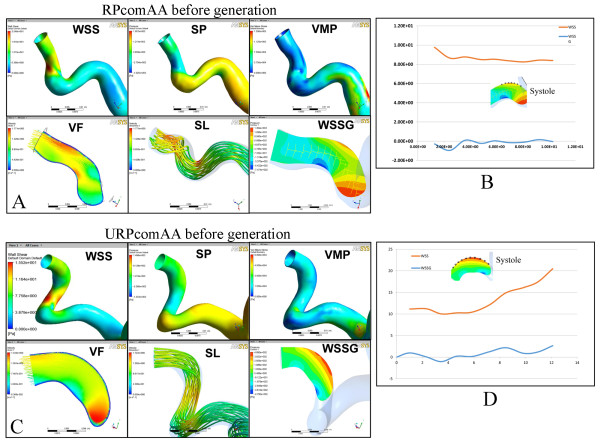
**Hemodynamic analyses of the RPcomAA and URPcomAA at the pre-aneurysm status. A**, Hemodynamic analyses in the RPcomAA and URPcomAA at the pre-aneurysm status in terms of WSS, pressure, velocity field, streamlines, and WSSG. **A**, **C**, elevated WSS, low pressure, high blood-flow velocity, and obvious changes in WSSG are observed at the site of aneurysm formation of the parent artery in the RPcomAA and URPcomAA models; **B**, **D**, Quantification along the longitudinal section of the area of aneurysm formation shows changes in the distribution of WSS and WSSG at the peak of the systolic period in both models. WSS, wall shear stress; P, pressure VMP, von Mises pressure; VF, velocity field; SL, streamlines; WSSG, wall shear stress gradient.

We observed elevated WSS and blood-flow velocity at the region of aneurysm formation compared with the adjacent parent artery in the RPcomAA (WSS, 17.7 ± 1.7 *vs*. 4.6 ± 1.2 Pa; velocity, 1312 ± 104.5 vs. 651.2 ± 133.3 mm/s), and a similar finding was observed in the URPcomAA (WSS, 12.1 ± 2.1 *vs*. 3.6 ± 0.4 Pa; velocity, 1111 ± 202.4 *vs*. 600 ± 56.2 mm/s). Additionally, the area of aneurysm formation was also subjected to a lower pressure compared with the adjacent parent artery in the RPcomAA (−30.7 ± 344.1 *vs*. 1418 ± 38.7 Pa) and URPcomAA (28.4 ± 201 vs. 883 ± 129.4 Pa). Observation of the velocity field and streamlines also showed turbulent blood flow in the area of aneurysm formation compared with the adjacent parent artery, which had ordered blood flow. A more significant change in WSSG was observed in the area of aneurysm formation in both models (Figures 4B and 5).

## Discussion

We used a vessel surface repair method to retrospectively undertake a hemodynamic study in a RPcomAA and URPcomAA before and after aneurysm formation. We had two main findings. Firstly, the RPcomAA and URPcomAA were subjected to the highest WSS at the distal aneurysm neck and decreased WSS at the aneurysm dome. However, the RPcomAA had a more complicated pattern of blood flow in the aneurysm sac, with formation of narrowed inflow jet and impaction zone. Secondly, hemodynamic analyses at pre-aneurysm status showed that the area of aneurysm formation of the parent artery was subject to extremely high WSS, elevated blood velocity, and WSSG changes compared with the adjacent arterial wall, in both models.

Ruptured aneurysms are usually larger than unruptured aneurysms, often with a mean diameter >7 mm in the posterior communicating aneurysm [1]. Non-dimensional parameters are thought to better reflect the geometric characteristics of an aneurysm than dimensional parameters, of which H/N and D/N are believed to be sensitive non-dimensional parameters [8,10]. H/N has also been proposed as an important parameter for predicting the likelihood of aneurysm rupture. Ujiie et al. studied 129 ruptured aneurysms and 78 unruptured aneurysms. They found no ruptured aneurysms with an H/N of <1.0, and almost 80% of the ruptured aneurysms showed an H/N of >1.6 [11,12]. In our study, H/N in the RPcomAA was 1.77 and it was 1.00 in the URPcomAA, which are consistent with the data of Ujiie et al.

In recent years, CFD studies of aneurysms and parent arteries have become an important method for investigating the mechanism of aneurysm formation and risk factors for rupture. A brain aneurysm is associated with arterial wall deficiencies, and hemodynamic studies of cerebral arteries at specific sites have revealed high WSS to be an important hemodynamic parameter leading to the formation or development of aneurysms. If blood flow increases, mechanical receptors in endothelial cells sense the tension of the vessel and respond by dilating the artery to reduce elevated WSS to normal levels (often 15–20 dyne/cm^2^) [13-16]. Another possible mechanism might be consistently high WSS to the arterial wall and the elastic layer leading to damage to endothelial cells and remodeling of the arterial wall, which may also explain why the highest WSS often presents at the aneurysm neck [7,16]. With regard to hemodynamic changes in the aneurysm sac, low WSS is often observed at the aneurysm dome, which may be closely related to the growth and rupture of aneurysms. This is because decreased WSS is likely to induce degeneration or an inflammation reaction in the aneurysm wall, which can potentially involve increased infiltration of inflammatory cells; activity of matrix metalloproteinases (MMPs); protein synthesis in the extracellular matrix; apoptosis of smooth muscle cells [17]. Upon comparison of WSS and WSSG at the aneurysm neck, body and dome, we found that the distal aneurysm neck was subject to the highest WSS in the RPcomAA and URPcomAA, whereas WSS at the aneurysm dome in the RPcomAA was lower than that observed in the URPcomAA. Changes in WSSG at the aneurysm dome in the RPcomAA were also more obvious than that in the URPcomAA. More importantly, a reverse study *via* surface repair of the diseased artery revealed that the area of aneurysm formation was subject to high WSS and apparent WSSG changes in both models, findings that are consistent with results from prospective observation studies [7].

Besides WSS, the pattern of blood flow also plays an important part in the growth and rupture of aneurysms. The complexity and stability of flow were divided into four flow types by Cebralet et al. [9]. The first two types are defined as “steady flow” and often present in unruptured aneurysms, whereas the latter two types are defined as “unsteady flow” and often occur in ruptured aneurysms, and have a close relationship with the growth and rupture of aneurysms [9]. An inflow jet and impaction zone are two specific flow indexes which have a close relationship with aneurysm rupture [9,18]. In general, a narrowed inflow jet or impaction zone are found in 80% and 76% of ruptured aneurysms, respectively. However, 82% of unruptured aneurysms may not have a narrowed inflow jet, and 75% have a wide impaction zone. For aneurysms with a narrowed inflow jet and narrowed impaction zone, the chance of rupture is 6.3-fold higher than those without a narrowed inflow jet or only a wide impaction zone [9]. In our cases, the flow pattern in the RPcomAA was type III, and was characterized with change in direction of the inflow jet and extremely narrowed impaction zone in the aneurysm sac. In the URPcomAA, a type-I flow pattern with a wide impaction zone was found. Our findings confirmed that aneurysms with different blood flow patterns in the sac may increase the risk of rupture. However, besides patterns of blood flow and WSS, degeneration of the extracellular matrix in the arterial wall by upregulation of MMP2 and MMP9 or infiltration of macrophages also have important synergistic effects with hemodynamic status if an aneurysm develops or ruptures [19,20].

Data modeling of the target vessel is a basic step for hemodynamic analyses. It also enables a diseased arterial wall to recover before aneurysm formation. Vasculature geometry as well as blood pressure, blood-flow velocity, and blood components in patients are similar before and after aneurysm formation, so the diseased arterial wall could be repaired by removing the aneurysm sac using a mesh repair method. Precise recovery of the diseased artery is an important method. We used the variance and mean curvature calculation method to determine the filling data in the absent area. This method enables calculation of filling data automatically based on the complex of the boundary contour of the data model. Then, the calculated data in the affected area can be used to construct a continuous surface that approximates to the original surface [21,22]. Lastly, the constructed surface can be used to choose different points for the measurement of hemodynamic parameters. Based on this theory, the hemodynamic parameters of points measured at the constructed surface provide full consideration of the effect of surrounding data.

The main deficiency of this study was that all comparisons were made based on hemodynamic analyses. However, the occurrence, development or rupture of aneurysms (especially brain aneurysms) is dependent upon genetics, degeneration of the arterial wall, hemodynamics, age, sex, smoking history, hypertension and artherosclerosis [5,15,23]. The second limitation was that we selected only two typical posterior communicating aneurysms for analyses. Future studies to include more aneurysm samples at different locations and multifactorial analyses should be conducted to better understand the generation or rupture of brain aneurysms.

## Conclusion

Hemodynamic analyses in patients with ruptured or unruptured aneurysms employing methods to simulate the situation before and after aneurysm formation using the vessel surface repair method are feasible, economical, and simple. Our preliminary results indicated that the arterial wall was subjected to elevated WSS, WSSG and blood-flow velocity before aneurysm generation. However, more complicated flow patterns (often with a narrrowed inflow jet or impaction zone) were more likely to be observed in ruptured aneurysms.

## Competing interests

We declare that we have no conflicts of interest in this article.

## Authors’ contributions

Study concept and design: ZYQ and LMH; Acquisition of data: LWJ, YL, XSM, THQ, ZYQ and CYS; Analysis and interpretation of data: LWJ and ZYQ; Drafting of the manuscript: LWJ; Critical revision of the manuscript for important intellectual content: LMH and CYS. All authors read and approved the final manuscript.

## Pre-publication history

The pre-publication history for this paper can be accessed here:

http://www.biomedcentral.com/1471-2377/13/166/prepub

## Supplementary Material

Additional file 1 Pre-processing setting of the conditions before hemodynamic analyses. Click here for file
