# Supplementary material for: New method for retrospective study of hemodynamic changes before and after aneurysm formation in patients with ruptured or unruptured aneurysms
Source: BMC Neurol. 2013 Nov 6;13:166. doi: 10.1186/1471-2377-13-166 (PMC4228259; doi:10.1186/1471-2377-13-166)
Supplement: Additional file 1 — Pre-processing setting of the conditions before hemodynamic analyses. [file 1471-2377-13-166-S1.doc]

**SUPPLEMENTAL MATERIAL**

**Supplementary Methods**

***CFD analysis***

For the solid component, the sweep method was used to build the hexahedral mesh. For the fluid component, the global size control and inflation method was used to build a transitional penta mesh between the tetra and hex mesh. Blood flow density (p) and viscosity (μ) were chosen as 1060 kg/m^3^ and 0.004 Pa/s, respectively. The vessel wall was idealized as rigid, and the no-slip boundary condition was applied to the walls of all models. We used orthogonal isotropic material to simulate the arterial wall, with density (p), Young’s modulus (E), and Poisson's ratio (ν) of 1060 kg/m^3^, 4.5e8 pa, and 0.3, respectively. The boundary conditions were set as follows. For the solid part, both ends of the vessel were fixed to set the fluid-solid interaction conditions. For the liquid part, the average blood flow velocity at one cardiac cycle was measured to define the inflow velocity curve of the model. To increase the convergence, the boundary condition at outflow was set as open.
